# Attentional and non-attentional systems in the maintenance of verbal information in working memory: the executive and phonological loops

**DOI:** 10.3389/fnhum.2014.00900

**Published:** 2014-11-07

**Authors:** Valérie Camos, Pierre Barrouillet

**Affiliations:** ^1^Laboratory of Cognitive Development, Fribourg Center for Cognition, Département de Psychologie, Université de FribourgFribourg, Switzerland; ^2^Developmental Cognitive Psychology, Faculté de Psychologie et des Sciences l’Education, Université de GenèveGenève, Switzerland

**Keywords:** working memory, attention, phonological loop, executive loop, rehearsal, refreshing

## Abstract

Working memory is the structure devoted to the maintenance of information at short term during concurrent processing activities. In this respect, the question regarding the nature of the mechanisms and systems fulfilling this maintenance function is of particular importance and has received various responses in the recent past. In the time-based resource-sharing (TBRS) model, we suggest that only two systems sustain the maintenance of information at the short term, counteracting the deleterious effect of temporal decay and interference. A non-attentional mechanism of verbal rehearsal, similar to the one described by Baddeley in the phonological loop model, uses language processes to reactivate phonological memory traces. Besides this domain-specific mechanism, an executive loop allows the reconstruction of memory traces through an attention-based mechanism of refreshing. The present paper reviews evidence of the involvement of these two independent systems in the maintenance of verbal memory items.

## Introduction

Working memory is a system dedicated to the storage and maintenance of information. This is a central system that allows us to interpret and comprehend our environment and ourselves by constructing transient representations. These representations are built using our internal and external states, and they can be conceived as mental models, as in Johnson-Laird’s (1983) theory. The role of working memory is to maintain these representations in face of decay and interference in order to avoid their loss, as well as to transform them for actions in accordance with our goals.

In the past decade, we have proposed a new model of working memory, named the Time-Based Resource-Sharing (TBRS) model (Barrouillet et al., 2004; see Barrouillet and Camos, 2012, 2015, for reviews). The TBRS model aims at accounting for complex span-related phenomena and more generally for working memory structure and functioning. Specifically, the TBRS model enlightens the role of attention in working memory by introducing the idea that attention is involved in the maintenance as well as in the processing of information. Moreover, our model allows an understanding of how attention is shared in a time-based manner between processing and maintenance, which are the two functions of working memory. Nevertheless, the TBRS model does not imply that attention is always involved in the maintenance activity of information in working memory. The aim of this review is to show that attentional and non-attentional systems subserve storage in working memory. Interestingly, these two types of systems are implicated in the maintenance of verbal information. After presenting these two maintenance systems within the framework of the TBRS model, we review evidence of the independence of these two systems, of their joint use to store verbal information, of the ability for adults to make adaptive choice between these systems, and of the impact that using one or the other system has on recall performance. We end our review with the presentation of some brain imaging data showing that different brain networks are distinguishable and would sustain each of these two maintenance systems.

## The maintenance of verbal information in the TBRS model

**Figure 1:**
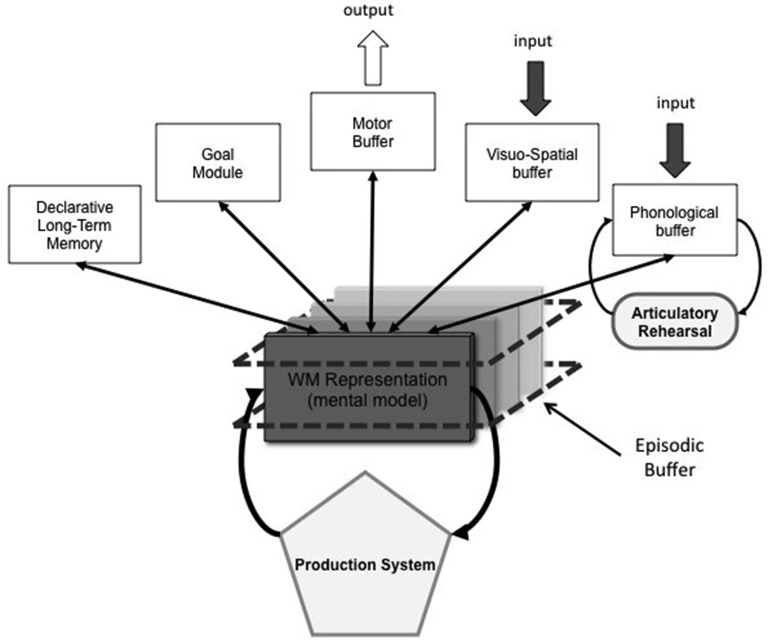
**The Time-Based Resource-Sharing model architecture (Figure 6.1 in Barrouillet and Camos, 2015, p. 118)**.

According to the TBRS model, two systems can be involved in the maintenance of verbal information (Figure 1). The first system we call the *executive loop* includes an episodic buffer and a procedural system. Similar to the episodic buffer in the multi-component model (Baddeley, 2000), we assume that working memory representations are stored in a buffer in which they suffer from temporal decay and interference, and consequently must be reconstructed or reactivated to permit any processing. Like in Adaptive Control of Thought—Rational (ACT-R) model (Anderson, 1993, 2007), the procedural system reads this representation and, depending on the currently active goal, maintains or updates its content by firing the appropriate production rule. The current goal can also command a switch to another of the working memory representations held in the episodic buffer. When the representations need to be maintained, their reconstruction is achieved through attentional refreshing which requires attention. This idea of an attention-based mechanism of maintenance is inspired by Cowan’s suggestion that memory items can be reactivated by a scanning process (Cowan, 1992; Vergauwe and Cowan, in press) or by the recirculation of items through the focus of attention (Cowan, 1995). However, although the TBRS model focuses on the role of attentional processes in working memory, the maintenance of verbal information could also be achieved through an articulatory rehearsal process as Baddeley described in his model of the *phonological loop* (Baddeley, 1986; Baddeley and Logie, 1999). Thus, in the TBRS model, the executive loop and the phonological loop are the two loops in charge of verbal maintenance. The functioning of the phonological loop had been extensively studied in the past 50 years (e.g., Baddeley, 2007). We now briefly outline the well-known effects emerging from its functioning, which will be discussed through this review as well. The phonological loop is involved in the storage of verbal information in a phonological format. As a consequence, the storage of phonologically similar words leads to more confusion than of dissimilar words. This effect is named the phonological similarity effect. To actively maintain memory traces in the phonological loop, memory items are subvocally rehearsed through processes shared by language production. The existence of such a verbal maintenance mechanism had two consequences. First, a concurrent articulation using similar language processes could block or at least impede the subvocal rehearsal of memory items and impair working memory recall. Second, lists of short words are better recalled than lists of long words, because in a fixed duration, the former take a shorter time to articulate and would consequently benefit from more rehearsals than the latter, increasing the probability to be recalled. This effect is named the word length effect.

Besides the phonological loop, the TBRS model describes another loop, the executive loop. Its functioning is based on four main proposals. First, the model assumes that both the processing and the maintenance of information within the executive loop rely on the same limited resource, which is attention. Because attention is a limited resource, it has to be shared between processing and storage. The second assumption is that many of the elementary cognitive steps involved in both processing and maintenance can only take place one at a time. When the executive loop is occupied by some processing episode, it is not available for the maintenance of memory items. The same idea is captured by the concept of the central bottleneck, developed by Pashler (1998), according to which central processes like response selection can only take place one at a time in such a way that the subsequent processes are postponed. Another way to express the same idea is to assume that the size of the focus of attention is limited to only one element with the consequence that attention could only select one item of knowledge at a time for the next cognitive operation (McElree and Dosher, 1989; Garavan, 1998; McElree, 1998, 2001; Oberauer, 2002, 2005). Thus, processing and storage activities could not take place in parallel, but alternate in occupying the central bottleneck. However, according to the third assumption of the TBRS model, as soon as attention is switched away, or in other words as soon as the focus of attention leaves the memory traces, their activation suffers from a time-related decay. Thus, in working memory span tasks, the memory traces of the to-be-maintained items decline when attention is occupied by the processing of distractors. Redirecting the focus of attention on the memory traces results in their refreshment before complete disappearance. Finally, the fourth assumption is that, due to the limitation of attention to only one element at a time and the time-related decay of memory traces outside the focus of attention, the sharing of attention is achieved through a rapid and incessant process of switching of this focus from processing to maintenance. As it can be suspected, most tasks typically do not induce a continuous capture of attention, and thus attention can be diverted from time to time, even for short periods of time, towards other thoughts and brought back to the current activity. This continuous switching of attention must be considered as a basic mental process underlying our phenomenal experience of thinking, which permits the coherence and cohesion of our mental life beyond the succession of changing thoughts. This conception in turn delineates a conception of cognitive load (CL). According to the TBRS framework, CL is the proportion of time during which tasks capture attention, thus impeding maintenance activities that require the executive loop. Within this framework, the CL is defined as: CL = Duration of attentional capture/Total time allowed to perform the task. It is important to note that even simple activities such as reading digits or response selection tasks can efficiently block attention for prolonged periods of time if they are performed under time constraints.

**Figure 2:**
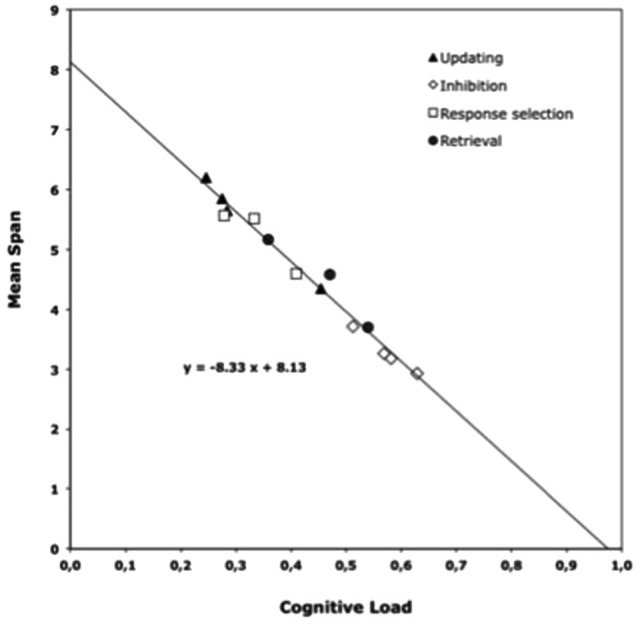
**Mean span (i.e., the mean number of maintained items) as a function of the cognitive load (Barrouillet et al., 2011)**.

The TBRS model and more specifically its conception of CL were verified in several studies (e.g., Barrouillet et al., 2004, 2007). For example, Barrouillet et al. (2011) asked adult participants to perform complex span tasks in which they had to maintain series of letters, digits or words of ascending length while performing various concurrent tasks. These concurrent tasks were a location or parity judgment tasks, a stroop task (classic and numerical versions), or a memory updating task (the 2-back task). Although these tasks differed in their nature, they are all well-known for capturing attention because they involve some response selection, retrieval, updating, or inhibition of information. All of these processes are considered executive functions and thus are highly attention-demanding. The mean response time when the answer was correct was used to estimate the duration of attentional capture of these tasks, and to compute their respective CL. As depicted in Figure 2, the number of memory items that can be maintained (expressed in mean span) while performing a concurrent task is a direct function of the CL of this task. More specifically, the mean span decreases linearly with the increase in CL, independently of the nature of the tasks and of the attention-demanding processes they involve. Several other studies replicated this finding with different types of memoranda (verbal, visual, or spatial), with various distracting activities, and in adults as well as in children and adolescents (Barrouillet and Camos, 2015, for a review). Such a finding strengthens the assumption made by the TBRS model that memory items can be maintained by the executive loop through attentional refreshing, which competes for attention with other attention-demanding processes required by working memory span tasks.

Although the effect of CL on recall performance appears even under concurrent articulation, one can wonder if the attentional refreshing is really different from the subvocal rehearsal described in Baddeley’s (1986) multi-component model. Using a reading span task in which participants had to maintain words while reading sentences presented in successive segments, Hudjetz and Oberauer (2007) manipulated the reading instructions in such a way that subvocal rehearsal was more or less impeded. For this purpose, participants had to read the sentences either continuously or at their own pace, the former condition strongly impeding the use of subvocal rehearsal. Moreover, the CL of the reading task was increased by increasing the pace of presentation of the segments to be read, with a fast pace shortening the time to perform the task and reducing the availability of attention for maintenance activities relative to a slow pace. If the maintenance of words relies only on attentional refreshing, recall performance should not be affected by the type of reading (i.e., continuously or at one’s own pace). Instead, only the pace of presentation would affect recall performance, with poorer recall at a fast than a slow pace. On the contrary, if the maintenance is achieved through subvocal rehearsal, the pace of presentation and the type of reading should interact. That is, when reading at their own pace, a slow pace of presentation would give participants more time to rehearse words. Conversely, the continuous reading would make rehearsal more difficult and the beneficial effect of the longer presentation at the slow pace would disappear. Although the results revealed a significant effect of both factors, the lack of interaction between pace of presentation and reading instructions contradicts the idea that maintenance relies exclusively on subvocal rehearsal. These results showed that a maintenance mechanism different from subvocal rehearsal is implicated in the maintenance of memoranda in working memory. Moreover, they gave the first support to the dissociation between attentional refreshing and subvocal rehearsal and, by extension, between the phonological loop and the executive loop.

To summarize, the TBRS model suggests the existence of two distinct systems of maintenance in working memory. One, the executive loop, is a domain-general system in which the attentional refreshing maintains any type of information (e.g., verbal, visual, multimodal) in an episodic buffer. The other, the phonological loop, is a domain-specific system dedicated to the maintenance of verbal information under phonological code and that does not require attention, at least after a brief initial setup period (Naveh-Benjamin and Jonides, 1984). Thus, these two systems differ on the type of representations they process as well as on their maintenance mechanism, and as a consequence on the implication of attention. These differences lead to several predictions. The existence of two systems implies they can be used independently from each other to maintain information, but also that they can be used jointly. Moreover, it can be suggested that participants could favor one or the other system of maintenance depending on some constraints or instructions. Finally, the well-known effects of phonological similarity and word length, specific to the phonological nature of the representations, should emerge when the phonological loop is involved in maintenance, but should not be affected by any variation in the implication of the executive loop. In the following, we present some experimental evidence supporting these different predictions.

## The independence of attentional and non-attentional maintenance systems

In a first study including four different experiments, Camos et al. (2009) tested the independence of the two loops hypothesized by the TBRS model. Using complex span tasks, the authors varied the opportunity of using one of the two loops while the other was impeded. For example, the phonological loop was impeded by a concurrent articulation whereas the availability of the executive loop was varied by introducing a more or less attention-demanding distracting task. As expected, the manipulation of the availability of one system while the other was impeded resulted in a reduction of recall performance, although participants were able to recall a decent number of verbal memory items. These findings confirmed the idea that both the executive and the phonological loops are able to maintain verbal information in working memory.

**Figure 3:**
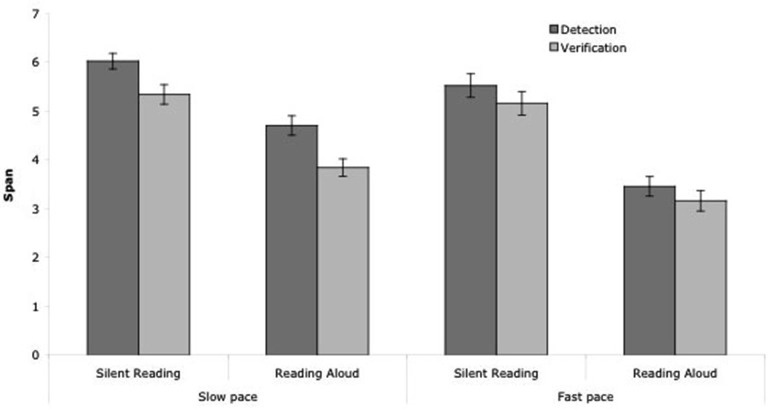
**Mean span according to the task (detection vs. verification of additions), the type of reading (silent vs. aloud) and the pace in Camos et al.’s (2009) Experiment 4**.

In further experiments, the interplay of these two systems was assessed by orthogonally manipulating the availability of attention and of articulatory processes. For example in Camos et al.’s (2009) Experiment 4, participants performed a distracting task that varied in attentional demand either silently or aloud. In a complex span task, each memory item was followed by a series of six digits successively displayed on screen. Participants had either to press the space bar when “5” appeared on screen (i.e., low-demanding detection task) or to verify if the 3rd and the 6th digits were the sum of the two previously presented digits (i.e., high-demanding verification task). These two experiments replicated the previous findings: impeding one or the other loop led to a reduction in recall performance. More interestingly, the effect of concurrent articulation was additive to the effect of the attentional demand resulting from the processing component of the complex span tasks (Figure 3). These results suggest that the phonological loop and the executive loop are two independent mechanisms involved in the maintenance of verbal information.

Camos et al.’s (2009) results are in line with the architecture proposed by the TBRS model with two distinct loops involved in the maintenance of verbal information. The existence of a second system, over and beyond the phonological loop and its rehearsal mechanism was initially mentioned by Baddeley and Hitch (1974), although never formally implemented in Baddeley’s multi-component model. This idea regularly reappeared within the multi-component model to explain how participants were able to maintain a substantial number of verbal items even under concurrent articulation (e.g., Vallar and Baddeley, 1982; Salamé and Baddeley, 1986; Hitch et al., 1989, 1993; Towse et al., 1998). Although the idea of different mechanisms intervening in the maintenance of verbal information could be found before in the literature, Camos et al.’s (2009) study provided the first empirical evidence of the independence of two maintenance systems, i.e., the executive and phonological loops.

## The joint use of the attentional and non-attentional systems

The existence of the two independent systems logically implies that they can be jointly used to maintain verbal items. Camos et al. (2009) provided some evidence of this joint use, as the introduction of concurrent articulation or concurrent attentional demand have an additive effect on recall performance, thereby supporting the idea that the two loops can act in conjunction. Another way to show the joint use of the phonological and executive loops was to examine the effects that the maintenance of verbal memory items has on concurrent processing activities.

Within the executive loop, when processing and storage are performed concurrently, processing episodes are postponed by maintenance activities in the same way that maintenance activities are postponed by processing. We have seen that the postponement of maintenance activities results in memory loss and thus reduced recall performance. However, the postponement of processing by maintenance activities should have a negligible effect on processing accuracy as long as the stimuli to be processed remain available in the environment. For example, this is the case in complex span tasks in which distractors remain on screen until the participant gives her response. Conversely, this postponement should appear in response times. Because attentional maintenance in the executive loop proceeds in a cumulative fashion, starting from the first list item and proceeding in forward order until the end (McCabe, 2008), this postponement should linearly increase with the number of memory items to be maintained. By contrast, when memory items are maintained within the phonological loop, such a postponement should not be observed.

To test this new set of predictions, Vergauwe et al. (2014) developed a new paradigm. They used a Brown-Peterson paradigm in which participants had to maintain a list of items for further recall and to perform an intervening task over a fixed retention interval prior to recall. However, participants were instructed to perform this intervening activity in such a way that, while trying to achieve the best performance in this task, they should not forget the memoranda. For example, in one experiment, participants were presented with series of 0 to 7 letters to be remembered, and asked during a 12-s retention interval to judge the parity of as many numbers as they can by pressing keys, each key press displaying a new number on screen. We assumed that to minimize the risk of forgetting and achieve a perfect recall of the memoranda, participants should refresh all of them before each processing episode.

**Figure 4:**
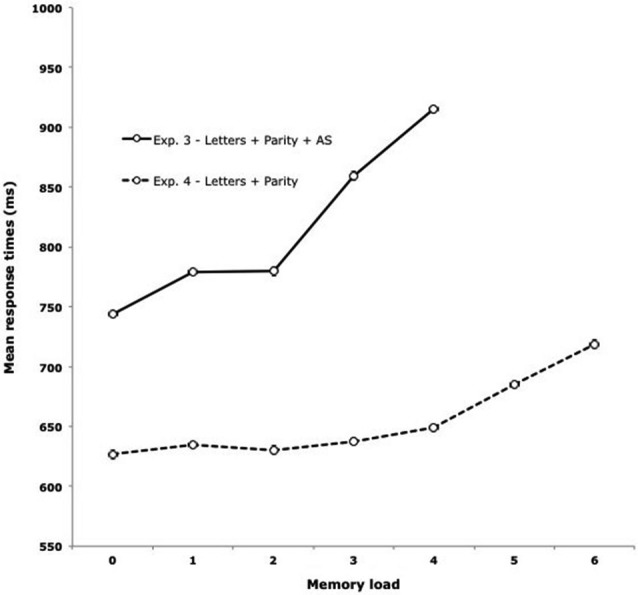
**Mean processing times in ms for letters as a function of memory load depending on the presence (Letters + Parity + AS, Experiment 3) or absence (Letters + Parity, Experiment 4) of articulatory suppression (AS) in Vergauwe et al. (2014)**.

In this experiment, to be sure that verbal information was only maintained through the executive loop, participants repeated “badibu” during the retention interval while completing the parity judgment task by pressing keys. Maintaining an increasing amount of verbal memoranda under concurrent articulation slowed the responses in the concurrent task (Figure 4, Experiment 3). The fact that refreshing memory items postpones concurrent processing activities indicates that the two activities compete for a general-purpose system. Contrary to attentional refreshing, the maintenance of verbal items through subvocal rehearsal requires very little attentional demand. Releasing the constraint to repeat “badibu” during the retention interval should allow participants to maintain as many verbal items as they can through subvocal rehearsal. As a consequence, attention would be available for performing the concurrent task without any postponement. This was the aim of Vergauwe et al.’s (2014) Experiment 4, in which participants had to maintain series of 0 to 7 letters while performing the parity judgment task, but contrary to the previous experiment, without any concurrent articulation. As illustrated in Figure 4, this experiment led to a very different pattern of results compared with Vergauwe et al.’s (2014) Experiment 3. Whereas the processing times steadily increased with the memory load under concurrent articulation, no such increase was observed in Experiment 4 till a load of four letters. As predicted, the slope remained nearly flat from 0 to 4 letters. This absence of postponement also contrasted with the increase in response times observed when memory load exceeded four letters. From 4 to 6 letters to be maintained, the slope was akin to the slope observed under concurrent articulation. Moreover, this slope gives an estimate of the speed of the refreshing process (around 50 ms per item). Such a value fits well with the neurophysiological explanation of short-term memory limitation by Lisman and Idiart (1995) in which each item would be stored in gamma oscillation subcycles (about 40 Hz) within a theta neuron network oscillation (see Luck and Vogel, 1998 for a similar account, and Vergauwe and Cowan, 2014, for discussion). These findings support the proposal made by the TBRS model of two distinct systems of verbal maintenance, with a verbal-specific system able to maintain up to four letters without any interference with a concurrent attention-demanding task, and an attentional system that competes with a concurrent task.

Apart from confirming the sequential functioning of working memory postulated by the TBRS model, these results shed light on the structure of working memory, confirming that there is a domain-general attentional system that is able to maintain verbal information and another non-attentional system that corresponds to the phonological loop in Baddeley’s (1986) theory.

## The adaptive choice between an attentional and a non-attentional systems

Another logical consequence of the existence of two independent systems is that adults should be able to favor one of them according to the constraints of the task or following instructions. To test the hypothesis that young adults can chose adaptively between the phonological loop and the executive loop, Camos et al. (2011) used a complex span paradigm in which the processing component was either a choice reaction time (CRT) task or a less demanding simple reaction time (SRT) task, and the memoranda were lists of six phonologically similar or dissimilar words. The rationale was that when the concurrent task is less demanding (i.e., SRT), attention would be available for maintenance through refreshing. Accordingly, participants should favor the executive loop because it likely enables the maintenance of non-phonological representations of the memory items. Such a mode of maintenance would reduce the confusability of the representations of the memoranda when they are phonologically similar words. By contrast, under a high attentional demand, participants should revert to the phonological loop, which requires less attention (Naveh-Benjamin and Jonides, 1984). Because subvocal rehearsal relies on the maintenance of phonological representations, recall performance in this case should suffer from the phonological similarity of some of the lists.

**Figure 5:**
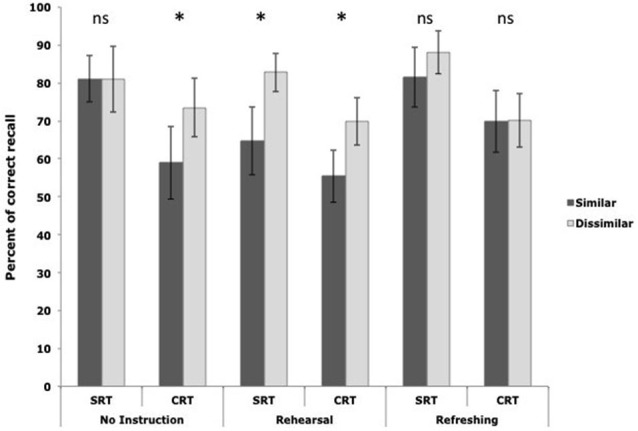
**Mean percentage of correct recall as a function of the phonological similarity of the memory words in a list (similar vs. dissimilar), the type of concurrent processing (SRT vs. CRT) and the maintenance mechanism participants were instructed to use in Camos et al. (2011)**.

As depicted in Figure 5 for the “no instruction” condition, when participants performed the CRT task, their recall was better for lists of phonologically dissimilar than similar words, replicating the phonological similarity effect. However, when participants performed the SRT task as a concurrent task, the phonological similarity effect disappeared. As predicted, the emergence of the phonological similarity effect depended on the attentional demand of the concurrent activity. The attention-demanding CRT task led participants to rely on subvocal rehearsal for maintenance, and the phonological similarity effect occurred. By contrast, the non-demanding SRT task allowed for the use of the executive loop, and thus the phonological characteristics of the memoranda did not affect recall. To verify that the change of pattern concerning the phonological similarity effect was due to a change in maintenance loop, two other groups of participants were instructed to perform the same complex span task while using either subvocal rehearsal or attentional refreshing to maintain series of words. As depicted in Figure 5 for rehearsal and refreshing instructions, whereas an increase in the attentional demand of the concurrent task led to reduced recall performance in both experiments, the occurrence of the phonological similarity effect depended on the instructions. Whatever the amount of attention available, the phonological similarity effect appeared when participants were instructed to use rehearsal. On the contrary, under refreshing instruction, the phonological characteristics of the lists to be maintained never affected recall performance. Thus, Camos et al. (2011) showed that the use of the two maintenance loops is adaptive and flexible, young adults being able to favor one of the loops according to its relative effectiveness or instructions. Moreover, these findings suggest that the phonological loop maintains verbal information under phonological representations as indexed by the emergence of a phonological similarity effect, and the executive loop acts probably on richer memory traces involving a variety of features, making recall performance immune to the phonological similarity of the memoranda in the lists.

## The attentional and non-attentional systems have different impact on recall

The aforementioned studies presented have made it clear that two systems of maintenance for verbal information exist, and have stressed their independence. Moreover, the TBRS model predicts that the use of one or the other of the two systems should have different effects on recall performance. Whereas any increase in CL of the processing component should impede the maintenance activities of the executive loop and thus reduce recall performance, the use of phonological loop should make recall susceptible to well-known effects specific to its functioning, i.e., the phonological similarity effect and the word length effect.

**Figure 6:**
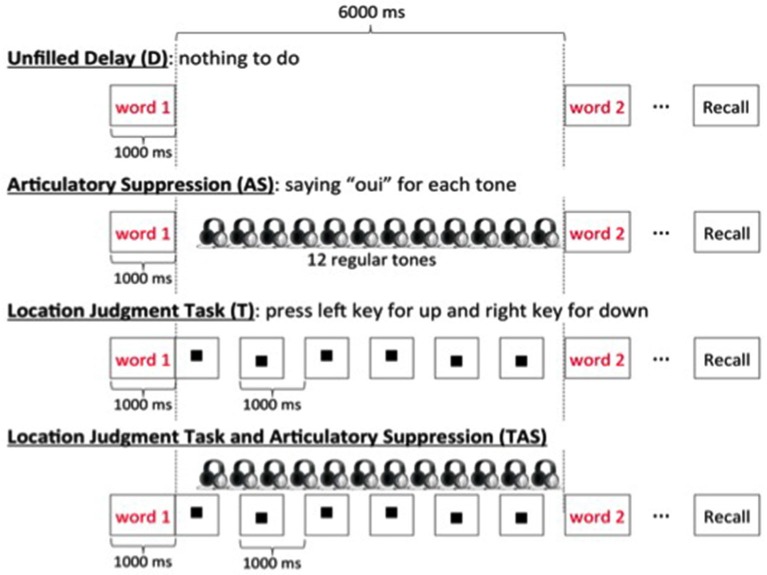
**The four conditions used in Camos et al. (2013) and in Mora and Camos (2013) in which each memory item was followed by an unfilled delay, by the repetitive utterance of “oui”, of a location judgment task, or the combination of the same location judgment task with the utterance of “oui”**.

To test these hypotheses, two studies orthogonally manipulated the attentional demand of the concurrent task and the availability of subvocal rehearsal (Camos et al., 2013; Mora and Camos, 2013). For this purpose, four complex span tasks were compared in which participants maintained series of either phonologically similar or dissimilar words, or series of short or long words (Figure 6). To vary the concurrent attentional demand, participants either performed a concurrent location judgment task or had nothing to do. The availability of subvocal rehearsal was varied either by allowing participants to remain silent through the concurrent task or by asking them to repeat the word “oui” (“yes” in French) at the rhythm of beeps heard in headphones. Both studies confirmed the predictions of the TBRS model.

**Figure 7:**
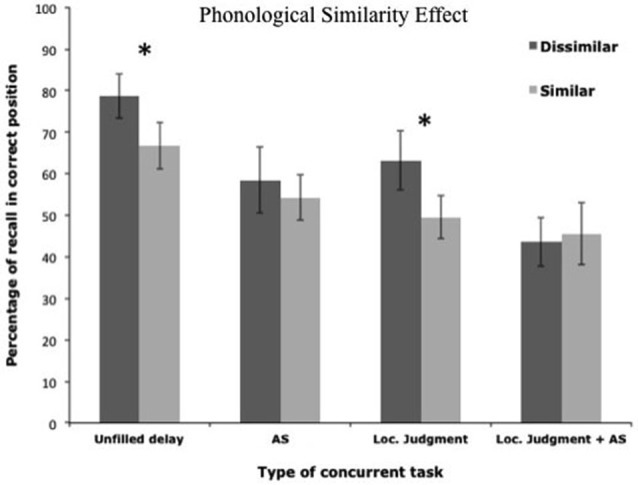
**Variation of the phonological similarity effect as a function of the four conditions used by Camos et al. (2013)**.

**Figure 8:**
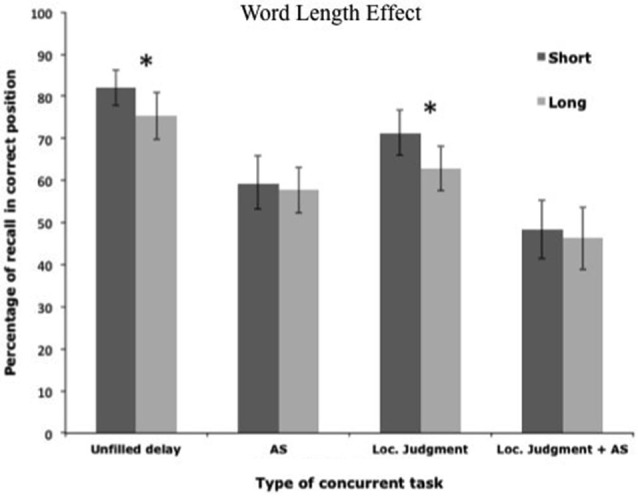
**Variation of the word length effect as a function of the four conditions used by Mora and Camos (2013)**.

The addition of a concurrent task or of a concurrent articulation resulted in reduced recall performance in both studies (Figures 7, 8). These studies also replicated the well-known phonological similarity effect with dissimilar word lists being better recalled than the similar word lists (Figure 7), as well as the word length effect with better recall performance for short than long words (Figure 8). However, these effects did not interact with the presence vs. absence of an attentional-demanding concurrent task, but disappeared under concurrent articulation. A similar disappearance of these effects was already reported in simple span tasks when the memory items were visually presented, and when a concurrent articulation occurred during the encoding of the memoranda (e.g., Coltheart, 1999; Fallon et al., 1999; Baddeley and Larsen, 2007). The present studies showed that blocking articulatory processes during maintenance could lead to the same disappearance of the phonological similarity and the word length effects as impeding the encoding processes. Thus, the impact of the phonological characteristics of the memory items depended on the use of the verbal-specific system, whereas recall performance was immune from these effects under the use of the executive loop.

## Distinct brain networks for the attentional and non-attentional maintenance systems

A vast amount of behavioral evidence supporting the distinction between a domain-general attentional system and a specialized non-attentional system was collected within the framework of the TBRS model. The literature brings further support to this distinction when examining the neural implementation of these two systems, and more specifically of their respective maintenance mechanisms, i.e., the attentional refreshing and the subvocal rehearsal.

Gruber (2001) observed that the brain network sustaining phonological storage is not uniquely localized, but depends on the possibility to rely or not on subvocal rehearsal. When a silent articulatory suppression prevents rehearsal, enhanced activity is observed in anterior prefrontal and inferior parietal brain areas. By contrast, when such suppression is relaxed, working memory performance activates Broca’s area and cortex along the left intraparietal sulcus. In other words, the non-articulatory maintenance of verbal information produced activation in a different network compared to subvocal rehearsal (see also Gruber and von Cramon, 2003, and Trost and Gruber, 2012, for similar findings). While these findings support the existence of two distinct systems of verbal maintenance, Raye et al. (2002, 2007) provided direct evidence of the dissociation between subvocal rehearsal and attentional refreshing, showing that they are neurally distinguishable processes. These authors showed an increased activation of the dorsolateral prefrontal cortex [DLPFC, Brodmann’s area (BA) 9] when young adults were instructed to refresh words (i.e., they were instructed to think briefly to the words) compared to repeating or reading them silently, or to simply press a button. Moreover, the results further distinguished attentional refreshing from subvocal rehearsal in revealing that Broca’s area (ventrolateral prefrontal cortex, VLPFC, BA 44) was selectively activated in a rehearsal condition (Raye et al., 2007). A similar finding was reported by Smith and Jonides (1999), suggesting that the use of subvocal rehearsal strategy relies on the activation of the VLPFC. Raye et al. (2007) concluded that the VLPFC reflects subvocal rehearsal of phonological information, while the DLPFC is assumed to reflect attention to various types of information (e.g., its activation did not differ between verbal and non-verbal information, Johnson et al., 2005). These neuroimagery data fit nicely with the TBRS model and the existence of two distinct systems. Indeed, whereas the Broca’s area is a specialized structure dedicated to language, the DLPFC is more broadly involved in executive control (D’Esposito et al., 1995). This neurological distinction between a specialized peripheral structure and an executive central structure echoes the differentiation introduced by the TBRS model between the phonological loop and the executive loop.

## Differences and commonalties with other working memory models

While the TBRS model proposes the existence of two distinct systems involved in the maintenance of verbal information at short term, in this section we examine other theoretical frameworks that either suggest similar systems or conversely advance alternative proposals. The model presenting the most obvious similarity with the TBRS model is the last version of the multi-component model (Baddeley, 2012). The multi-component model of working memory distinguishes a central system from a domain-specific system for verbal information (Baddeley, 1986, 2000; Baddeley and Logie, 1999). For the domain-specific system, both models propose that verbal information is maintained in a phonological store by verbal rehearsal through a phonological loop. Moreover, the executive loop described by the TBRS model includes an episodic buffer in which cross-domain representations are constructed and maintained as suggested by Baddeley et al. (2010). Besides these similarities, the TBRS theory departs from the multi-component approach primarily in the structure and functioning of the central component. Whereas its structure and functioning of the central executive remains underspecified in Baddeley’s theory, we have suggested that it can be conceived as an executive loop connecting the episodic buffer with a procedural system. As a consequence and contrary to Baddeley’s modal model, the central system in the TBRS model is in charge of both processing and storage activities that compete for a common supply. Nevertheless, as noted above, in several works framed within the multicomponent model, Baddeley and collaborators have suggested another system that would supplement the phonological loop when overloaded (e.g., Baddeley and Hitch, 1974; Salamé and Baddeley, 1986). However, the TBRS model specifies the main constraints of this central system that are due to the sequential functioning of the executive loop and the ephemeral nature of working memory representations. As explained above, these two characteristics are at the root of the relationship between storage capacity and concurrent attentional demand, as seen in Figure 2.

The existence of such a conflict between processing and storage is also predicted by Cowan’s (1999, 2005) embedded-processes model. Cowan assumes that working memory can be conceived as the temporarily activated portion of long-term memory, with a subset of this activated memory that corresponds to the focus of attention. The focus of attention is limited to three to five chunks of information (see Luck and Vogel, 1997, for similar estimate in visuospatial domain), and is controlled by automatic orienting responses to changes in the environment as well as voluntary effort directed by the central executive towards current goals. A consequence of this working memory structure is that the focus of attention is involved in both the retention of information and in processing activities. Congruent with our distinction between an executive and phonological loops, Cowan also stresses that the capacity of the focus of attention appears clearly when verbal rehearsal is prevented, suggesting that maintenance of verbal information through attentional focusing and verbal rehearsal must be distinguished.

Other theories assume that working memory is the activated part of long-term memory, such as the concentric model proposed by Oberauer (2002) who distinguishes, within this activated part, a region of direct access containing about four items. Among them, one item is selected by the focus of attention for processing (see also McElree, 1998, 2001). Within the TBRS model, it can be conceived that the single item within the focus of attention would correspond to the working memory representation currently processed by the executive loop, whereas the region of direct access corresponds to the representations held in the episodic buffer. Consequently, the diverging conceptions about the size of the focus of attention (i.e., either one or four items according to Oberauer or Cowan, respectively) can be reconciled by considering that the sequential functioning of the executive loop requires that several representations are almost simultaneously present to the mind (i.e., in the episodic buffer), while only one representation is currently refreshed (cf. Figure 1). However, neither Cowan’s nor Oberauer’s theories stress the importance of temporal factors as the TBRS model does.

All of the previously highlighted theories assume that working memory is concerned with those processes that require attention (see also Lovett et al., 1999). Engle’s model made this point very clear in distinguishing working memory from short-term memory by the implication of controlled attention in working memory tasks (Engle et al., 1999; Engle and Kane, 2004). This model suggests that working memory is mainly involved on those activities that need executive control to maintain goal-relevant information under conditions of interference or competition. The TBRS model departs partially from this view, because it does not limit the attention involved in working memory to the controlled or executive attention. Of course, working memory is important to maintain information in an active state and to solve the conflicts resulting from interference and activation of prepotent but inappropriate responses as Engle and Kane argue (e.g., Rosen and Engle, 1998; Kane and Engle, 2003; Unsworth et al., 2004; Bunting, 2006). However, attention is also important for simple activations of knowledge from long-term memory, as suggested by theories like ACT-R (Anderson, 1993). As reported previously, even the identification of material as simple as digits or of a location can capture attention for a sizable amount of time that it leads to a reduction of recall performance.

More recently, Unsworth and Engle (2007a,b) have also suggested that working memory comprises two functionally different components, although rather different from the two loops described in the TBRS model. A first component, referred to as primary memory, actively maintains information over the short term. According to Unsworth et al. (2010), this component is conceptually similar to Cowan’s focus of attention. The second component, referred to as secondary memory, is needed to retrieve information that can not be maintained in primary memory when its capacity is exhausted or its content is displaced by irrelevant distracters. In this case, retrieval from secondary memory would rely on a cue-dependent mechanism. In the case of complex span tasks as used in the experiments reported above, memory items would be first stored in primary memory, but quickly displaced into secondary memory by the processing activity. At recall, the majority of the items would have to be retrieved from secondary memory through strategic search. By contrast, in simple span tasks, items could be held in primary memory, at least when list lengths are small (i.e., up to four items). With longer lists, items would be initially held in primary memory but some of them would be displaced into secondary memory.

Although Unsworth and Engle (2007a) did not explicitly claim it, it could be imagined that what they call primary memory corresponds to the phonological loop because they focused on verbal memory. Indeed, Vergauwe et al. (2014) observed that this phonological loop can hold up to four items, and when this capacity is exhausted, the executive loop works as a back-up system to refresh working memory representations. This option was evoked by Jarrold et al. (2010) who suggested that primary memory capacity could be supported by rehearsal. However, our review has made it clear that the phonological loop can maintain verbal items without interfering with attention demanding activities, demonstrating that it is distinct from the focus of attention. As a consequence, the phonological loop can not stand as the primary memory described by Unsworth and Engle (2007a). Another possibility would be to consider that primary memory consists of the assembly of the phonological and the executive loops, with secondary memory corresponding to long-term memory. However, we have seen that when participants were free to use verbal rehearsal for maintaining letters, (i.e., when both the phonological and the executive loops could be used for maintenance purpose), they were able to maintain up to six letters while performing a distracting task, which is beyond the expected primary memory capacity.

Finally, it is noticeable that the TBRS model is inspired by the ACT-R architecture (Anderson, 1993, 2007; Anderson and Lebière, 1998) and its procedural system. Within ACT-R, an imaginal module is used for storing intermediate information necessary for performing tasks and is comparable with the focus of attention in Cowan’s (2005) theory or with Baddeley’s episodic buffer. This imaginal module or problem state resource would be limited to only one coherent chunk of information with three or four slots and would create interference when it is requested by more than one task (Borst et al., 2010). Though our proposals are akin to these conceptions, some differences remain. Indeed, Borst et al. (2010) specify that not all tasks require the use of the imaginal module, for example when no intermediary results need to be stored or when all the necessary information is present in the world. Our conception differs because we assume that any process requiring an executive function involves the executive loop. Indeed, our results make clear that a simple response selection associated with the location of a stimulus on screen, or the direct retrieval of parity information that does not require any intermediary result, compete with concurrent maintenance of information within the episodic buffer. This suggests a more general and central representational role for the episodic buffer and the executive loop in our model than for the imaginal module postulated by ACT-R.

## Conclusion

In summary, the two maintenance loops described in the TBRS model, the executive loop and the phonological loop, are two distinct and independent systems. Because of this independence, they can be jointly used to maintain verbal information. Moreover, adults can chose to favor the use of one or the other system, either intentionally when instructed to do so or adaptively because the use of the executive loop could reduce the confusion for phonologically similar material. However, the use of one or the other loop is not without consequence. Each of these systems of maintenance induces a different pattern of recall performance. The use of the phonological loop makes recall sensitive to the phonological characteristics of the material to be maintained, whereas the phonological nature of the memory items does not affect recall performance under the use of the executive loop. This does not imply that the executive loop is a “better” system of maintenance that should always be favored for verbal information. Because attentional refreshing is more attention-demanding than subvocal rehearsal, the former is very sensitive to the availability of attention and the presence of concurrent attention demanding task. Finally, brain imaging studies reported distinct neural structures supporting the separation of these two loops.

## Conflict of interest statement

The authors declare that the research was conducted in the absence of any commercial or financial relationships that could be construed as a potential conflict of interest.

## References

[B1] Anderson J. R. (1993). Rules of the Mind. Hillsdale, NJ: Lawrence Erlbaum Associates.

[B2] Anderson J. R. (2007). How Can the Human Mind Occur in the Physical Universe? New York, NY: Oxford University Press.

[B3] Anderson J. R. Lebière C. (1998). The Atomic Components of Thought. Mawhaw, NJ: Lawrence Erlbaum Associates.

[B4] Baddeley A. D. (1986). Working Memory. Oxford: Clarendon Press.

[B5] Baddeley A. D. (2000). The episodic buffer: a new component of working memory? Trends Cogn. Sci. 4, 417–423. 10.1016/s1364-6613(00)01538-2 11058819

[B6] Baddeley A. D. (2007). Working Memory, Thought and Action. Oxford: Oxford University Press.

[B7] Baddeley A. D. (2012). Working memory: theories, models and controversies. Ann. Rev. Psychol. 63, 1–29. 10.1146/annurev-psych-120710-100422 21961947

[B8] Baddeley A. D. Allen R. J. Hitch G. J. (2010). Investigating the episodic buffer. Psychol. Belg. 50, 223–243 10.5334/pb-50-3-4-223

[B9] Baddeley A. D. Hitch G. J. (1974). “Working memory,” in Recent Advances in Learning and Motivation, ed Bower G. A. (New York: Academic Press), 647–667.

[B10] Baddeley A. D. Larsen J. D. (2007). The phonological loop unmasked? A comment on the evidence for a “perceptual-gestural” alternative. Q. J. Exp. Psychol. (Hove) 60, 497–504. 10.1080/17470210601147572 17455059

[B11] Baddeley A. D. Logie R. H. (1999). “Working memory: the multiple-component model,” in Models of Working Memory: Mechanisms of Active Maintenance and Executive Control, eds Miyake A. Shah P. (Cambridge: Cambridge University Press), 28–61.

[B12] Barrouillet P. Bernardin S. Camos V. (2004). Time constraints and resource-sharing in adults’ working memory spans. J. Exp. Psychol. Gen. 133, 83–100. 10.1037/0096-3445.133.1.83 14979753

[B13] Barrouillet P. Bernardin S. Portrat S. Vergauwe E. Camos V. (2007). Time and cognitive load in working memory. J. Exp. Psychol. Learn. Mem. Cog. 33, 570–585. 10.1037/0278-7393.33.3.570 17470006

[B14] Barrouillet P. Camos V. (2012). As time goes by: temporal constraints in working memory. Curr. Dir. Psychol. Sci. 21, 413–419 10.1177/0963721412459513

[B15] Barrouillet P. Camos V. (2015). Working Memory: Loss and Reconstruction. Hove: Psychology Press.

[B16] Barrouillet P. Portrat S. Camos V. (2011). On the law relating processing and storage in working memory. Psychol. Rev. 118, 175–192. 10.1037/a0022324 21480738

[B17] Borst J. P. Taatgen N. A. van Rijn H. (2010). The problem state: a cognitive bottleneck in multitasking. J. Exp. Psychol. Learn. Mem. Cog. 36, 363–382. 10.1037/a0018106 20192536

[B18] Bunting M. F. (2006). Proactive interference and item similarity in working memory. J. Exp. Psychol. Learn. Mem. Cog. 32, 183–196. 10.1037/0278-7393.32.2.183 16569140

[B19] Camos V. Lagner P. Barrouillet P. (2009). Two maintenance mechanisms of verbal information in working memory. J. Mem. Lang. 61, 457–469 10.1016/j.jml.2009.06.002

[B20] Camos V. Mora G. Barrouillet P. (2013). Phonological similarity effect in complex span task. Q. J. Exp. Psychol. 66, 1927–1950. 10.1080/17470218.2013.768275 23419012

[B21] Camos V. Mora G. Oberauer K. (2011). Adaptive choice between articulatory rehearsal and attentional refreshing in verbal working memory. Mem. Cognit. 39, 231–244. 10.3758/s13421-010-0011-x 21264630

[B22] Coltheart V. (1999). Comparing short-term memory and memory for rapidly presented visual stimuli. Int. J. Psychol. 34, 293–300 10.1080/002075999399594

[B23] Cowan N. (1992). Verbal memory span and the timing of spoken recall. J. Mem. Lang. 31, 668–684 10.1016/0749-596x(92)90034-u

[B95] Cowan N. (1995). Attention and Memory: An Integrated Framework. New York, NY: Oxford University Press.

[B24] Cowan N. (1999). “An embedded-process model of working memory,” in Models of Working Memory: Mechanisms of Active Maintenance and Executive Control, eds Miyake A. Shah P. (Cambridge: Cambridge University Press), 62–101.

[B25] Cowan N. (2005). Working Memory Capacity. Hove: Psychology Press.

[B26] D’Esposito M. Detre J. A. Alsop D. C. Shin R. K. Atlas S. Grossman M. (1995). The neural basis of the central executive system of working memory. Nature 378, 279–281. 10.1038/378279a0 7477346

[B27] Engle R. W. Kane M. J. (2004). “Executive attention, working memory capacity and a two-factor theory of cognitive control,” in The Psychology of Learning and Motivation (Vol. 44), ed Ross B. (New York: Elsevier), 145–199.

[B28] Engle R. W. Kane M. J. Tuholski S. W. (1999). “Individual differences in working memory capacity and what they tell us about controlled attention, general fluid intelligence and functions of the prefrontal cortex,” in Models of Working Memory: Mechanisms of Active Maintenance and Executive Control, eds Miyake A. Shah P. (Cambridge: Cambridge University Press), 102–134.

[B29] Fallon A. B. Groves K. Tehan G. (1999). Phonological similarity and trace degradation in the serial recall task: when CAT helps RAT, but not MAN. Int. J. Psychol. 34, 301–307 10.1080/002075999399602

[B30] Garavan H. (1998). Serial attention within working memory. Mem. Cognit. 26, 263–276. 10.3758/bf03201138 9584434

[B31] Gruber O. (2001). Effects of domain-specific interference on brain activation associated with verbal working memory task performance. Cereb. Cortex 11, 1047–1055. 10.1093/cercor/11.11.1047 11590114

[B32] Gruber O. von Cramon D. Y. (2003). The functional neuroanatomy of human working memory revisited: evidence from 3-T fMRI studies using classical domain-specific interference tasks. Neuroimage 19, 797–809. 10.1016/s1053-8119(03)00089-2 12880808

[B33] Hitch G. J. Halliday M. S. Littler J. E. (1989). Item identification time, rehearsal rate and memory span in children. Q. J. Exp. Psychol. 41, 321–337 10.1080/14640748908402368

[B34] Hitch G. J. Halliday M. S. Littler J. E. (1993). Development of memory span for spoken words: the role of rehearsal and item identification processes. Br. J. Dev. Psychol. 11, 159–169 10.1111/j.2044-835x.1993.tb00595.x

[B35] Hudjetz A. Oberauer K. (2007). The effects of processing time and processing rate on forgetting in working memory: testing four models of the complex span paradigm. Mem. Cognit. 35, 1675–1684. 10.3758/bf03193501 18062545

[B36] Jarrold C. Tam H. Baddeley A. D. Harvey C. E. (2010). The nature and the position of processing determines why forgetting occurs in working memory tasks. Psychon. Bull. Rev. 17, 772–777. 10.3758/pbr.17.6.772 21169567

[B37] Johnson M. K. Raye C. L. Mitchell K. J. Greene E. J. Cunningham W. A. Sanislow C. A. (2005). Using fMRI to investigate a component process of reflection: prefrontal correlates of refreshing a just activated representation. Cogn. Affect. Behav. Neurosci. 5, 39–361. 10.3758/CABN.5.3.339 16396094

[B38] Johnson-Laird P. N. (1983). Mental Models. Cambridge: Cambridge University Press.

[B39] Kane M. J. Engle R. W. (2003). Working-memory capacity and the control of attention: the contributions of goal neglect, response competition and task set to stroop interference. J. Exp. Psychol. Gen. 132, 47–70. 10.1037/0096-3445.132.1.47 12656297

[B40] Lisman J. E. Idiart M. A. P. (1995). Storage of 7 +/− 2 short-term memories in oscillatory subcycles. Science 267, 1512–1515. 10.1126/science.7878473 7878473

[B41] Lovett M. C. Reder L. M. Lebière C. (1999). “Modeling working memory in a unified architecture: an ACT-R perspective,” in Models of Working Memory: Mechanisms of Active Maintenance and Executive Control, eds Miyake A. Shah P. (Cambridge: Cambridge University Press), 135–182.

[B42] Luck S. J. Vogel E. K. (1997). The capacity of visual working memory for features and conjunctions. Nature 390, 279–281. 9384378 10.1038/36846

[B43] Luck S. J. Vogel E. K. (1998). Response to visual and auditory working memory capacity by N. Cowan. Trends Cogn. Sci. 2, 78–80 10.1016/s1364-6613(98)01144-9 21227076

[B44] McCabe D. P. (2008). The role of covert retrieval in working memory span tasks: evidence from delayed recall tests. J. Mem. Lang. 58, 480–494. 10.1016/j.jml.2007.04.004 19633737 PMC2715014

[B45] McElree B. (1998). Attended and non-attended states in working memory: accessing categorized structures. J. Mem. Lang 38, 225–252 10.1006/jmla.1997.2545

[B46] McElree B. (2001). Working memory and focal attention. J. Exp. Psychol. Learn. Mem. Cogn. 27, 817–835. 10.1037//0278-7393.27.3.817 11394682 PMC3077110

[B47] McElree B. Dosher B. A. (1989). Serial position and set size in short-term memory: the time course of recognition. J. Exp. Psychol. Gen. 118, 346–373.

[B48] Mora G. Camos V. (2013). Two systems of maintenance in verbal working memory: evidence from the word length effect. PLoS One 8:e70026. 10.1371/journal.pone.0070026 23894580 PMC3722204

[B49] Naveh-Benjamin M. Jonides J. (1984). Maintenance rehearsal: a two-component analysis. J. Exp. Psychol. Learn. Mem. Cogn. 10, 369–385 10.1037//0278-7393.10.3.369

[B50] Oberauer K. (2002). Access to information in working memory: exploring the focus of attention. J. Exp. Psychol. Learn. Mem. Cogn. 28, 411–421. 10.1037//0278-7393.28.3.411 12018494

[B51] Oberauer K. (2005). Control of the contents of working memory: a comparison of two paradigms and two age groups. J. Exp. Psychol. Learn. Mem. Cogn. 31, 714–728. 10.1037/0278-7393.31.4.714 16060775

[B98] Pashler H. (1998). The Psychology of Attention. Cambridge, MA: MIT Press.

[B52] Raye C. L. Johnson M. K. Mitchell K. J. Greene E. J. Johnson M. R. (2007). Refreshing: a minimal executive function. Cortex 43, 135–145. 10.1016/s0010-9452(08)70451-9 17334213

[B53] Raye C. L. Johnson M. K. Mitchell K. J. Reeder J. A. Greene E. J. (2002). Neuroimaging a single thought: Dorsolateral PFC activity associated with refreshing just-activated information. Neuroimage 15, 447–453. 10.1006/nimg.2001.0983 11798278

[B54] Rosen V. M. Engle R. W. (1998). Working memory capacity and suppression. J. Mem. Lang. 39, 418–436 10.1006/jmla.1998.2590

[B55] Salamé P. Baddeley A. D. (1986). Phonological factors in STM: similarity and the unattended speech effect. B. Psychonomic Soc. 24, 263–265 10.3758/bf03330135

[B56] Smith E. E. Jonides J. (1999). Storage and executive processes in the frontal lobes. Science 283, 1657–1661. 10.1126/science.283.5408.1657 10073923

[B57] Towse J. N. Hitch G. J. Hutton U. (1998). A reevaluation of working memory capacity in children. J. Mem. Lang. 39, 195–217 10.1006/jmla.1998.2574

[B58] Trost S. Gruber O. (2012). Evidence for a double dissociation of articulatory rehearsal and non-articulatory maintenance of phonological information in human verbal working memory. Neuropsychobiology 65, 133–140. 10.1159/000332335 22378145

[B59] Unsworth N. Engle R. W. (2007a). On the division of short-term and working memory: an examination of simple and complex span and their relation to higher order abilities. Psychol. Bull. 133, 1038–1066. 10.1037/0033-2909.133.6.1038 17967093

[B60] Unsworth N. Engle R. W. (2007b). The nature of individual differences in working memory capacity: active maintenance in primary memory and controlled search from secondary memory. Psychol. Rev. 114, 104–132. 10.1037/0033-295x.114.1.104 17227183

[B61] Unsworth N. Schrock J. C. Engle R. W. (2004). Working memory capacity and the antisaccade task: individual differences in voluntary saccade control. J. Exp. Psychol. Learn. Mem. Cogn. 30, 1302–1321. 10.1037/0278-7393.30.6.1302 15521806

[B62] Unsworth N. Spillers G. J. Brewer G. A. (2010). The contributions of primary and secondary memory to working memory capacity: an individual differences analysis of immediate free recall. J. Exp. Psychol. Learn. Mem. Cogn. 36, 240–247. 10.1037/a0017739 20053060

[B63] Vallar G. Baddeley A. D. (1982). Short-term forgetting and the articulatory loop. Q. J. Exp. Psychol. 34, 53–60 10.1080/14640748208400857

[B64] Vergauwe E. Camos V. Barrouillet P. (2014). The impact of storage on processing: implications for structure and functioning of working memory. J. Exp. Psychol. Learn. Mem. Cogn. 40, 1072–1095. 10.1037/a0035779 24564542

[B65] Vergauwe E. Cowan N. (2014). A common short-term memory retrieval rate may describe many cognitive procedures. Front. Hum. Neurosci. 8:126. 10.3389/fnhum.2014.00126 24639643 PMC3945934

[B66] Vergauwe E. Cowan N. (in press). Attending to items in working memory: evidence that refreshing and memory search are closely related. Psychon. B. Rev. 10.3758/s13423-014-0755-6 PMC4417097 25361821

